# Evaluation of the efficacy of commonly used disinfectants against isolated chlorine-resistant strains from drinking water used in Egyptian cattle farms

**DOI:** 10.14202/vetworld.2019.2025-2035

**Published:** 2019-12-21

**Authors:** Mohamed Abdelhameed Kamal, Mahmoud Abdelaty Khalaf, Zakia Attia Mohamed Ahmed, Jakeen El Jakee

**Affiliations:** 1 Department of Veterinary Hygiene and Management, Faculty of Veterinary Medicine, Cairo University, Giza 11221, Egypt; 2 Department of Microbiology, Faculty of Veterinary Medicine, Cairo University, Giza 11221, Egypt

**Keywords:** cattle farms, disinfectant resistance, drinking water, microbial profile, resistance genes

## Abstract

**Background and Aim:**

Drinking water of poor microbiological quality contains high percentages of microbes causing outbreaks of mainly coliform-related diseases. These microbes could be controlled by many hygienic standards including disinfection, but disinfectants misuse causes the developing of disinfectant-resistant strains. The present study aimed to investigate drinking water bacterial profile, determine chlorine-resistant strains, and statistically correlate that with the used disinfectant and disinfection process variables. *In vitro* evaluation of the bactericidal effect of the most commonly used disinfectants in cattle operations against the isolated chlorine-resistant strains and detection of *qacE* resistance gene in the isolated chlorine-resistant *Escherichia coli* strains in some cattle farms suffering coliform and non-coliform related disease around Egypt.

**Materials and Methods:**

A structured questionnaire is used to survey a convenience sample of 132 Egyptian cattle beef and dairy farms suffering emerged epidemics to identify commonly used disinfection process, disinfectant types, disinfectants frequency, and rate of use. One hundred and thirty-two water samples were collected for microbiological analysis to obtain water bacterial profile and testing resistance to chlorine. Statistical analysis was performed to identify the level of association between microbial profile and presence of chlorine-resistant strains in each farm with used disinfection, disinfectant types, and rate of use in these farms.

**Results:**

A wide range of disinfectant types used for variable purposes inside cattle farms with a different frequency of use and the highest percent of farms 25.8% use 4-5 types of disinfectants, followed by 25% of farms use two types, then 18.9% use three types. Microbial profile of water samples revealed isolation of *E. coli*, *Streptococcus faecalis*, *Pseudomonas aeruginosa*, *Klebsiella* spp., *Proteus* spp., *Salmonella* spp., *Enterobacter* spp., *Citrobacter* spp., *Shigella flexneri*, *Serratia marcescens*, and *Yersinia enterocolitica* in percent (98.5, 97.7, 97.7, 76.5, 66.7, 36.4, 78.8, 74.2, 30.3, 29.5, and 14.4% of cattle farms, respectively), from which five *E. coli*, four *Salmonella*, four *Pseudomonas*, two *Klebsiella*, and four *Streptococcus* strains expressed chlorine resistance. Statistical analysis showed weak to moderate correlation (rho 0.15-0.46) between bacterial profile strains count and presence of resistant strains with different farm disinfection, disinfectant types, and rate of use. Experimental evaluation of the bactericidal effect of the eight selected disinfectants on the chlorine-resistant isolated strains revealed that peroxymonosulfate killed 19/19 isolated strains/15 min contact time, and quaternary ammonium compounds killed only 3/19 strains/15 min contact time. The *qacE* resistance gene was detected in 3/4 isolated chlorine-resistant *E. coli* strains.

**Conclusion:**

Drinking water microbial profile strains and resistance to disinfectants are widely varied in cattle farms, and this variance depends on critical factors among which the disinfection process types used disinfectant types and frequency of disinfectants use or change.

## Introduction

Water, as a critical nutrient, is second only to oxygen for keeping life and maximizes growth, lactation, and reproduction of bovine spp. The water needs per one unit of bovine body mass is higher than any other mammal. Seventy to 97% of water needed by cattle was from drinking water. Furthermore, drinking water quality is critical, as it affects cattle health and productivity. Water quality is determined by water source type and contamination level from abiotic and biotic origin which either dissolved nutrients or directly deposited urine or feces [1].

Quality of drinking water is evaluated by major aspects, among which, the water microbial profile and contaminants, which are among the most detrimental parameters reducing the drinking water quality [2]. The major bacteria found in polluted water, are coliform bacteria. The highly important species of the group include *Klebsiella* spp., *Enterobacter* spp., and *Escherichia coli*. Furthermore, non-coliform bacteria were isolated in polluted water such as *Proteus*, *Streptococcus*, *Salmonella*, and *Pseudomonas* species [3].

In cattle water troughs, the highest percentages of microbes causing outbreaks of coliform-related diseases, are *E. coli*, *Klebsiella*, and *Enterobacter aerogenes* species predisposing to diarrhea, mastitis, urinary infections, and other unsavory lethal infections, drinking water contaminated with manure became a nidus for bacterial growth leading to animal diseases. *E. coli* is critical as mastitis pathogens and highly distributed in the livestock farm environment and associated with gastrointestinal and extra-intestinal infections (e.g., septicemia, mastitis, and urinary tract infections) in both humans and animals [4]. *Klebsiella* has economic impact which is more devastating as many cows die or end up being culled, *Klebsiella* is usually referred to as particularly aggressive and is prone to cause severe clinical mastitis, which responds poorly to treatment and as a consequence, infections tend to be severe and long-lasting with a fatal outcome, in the etiology of bovine mastitis, Gram-negative organisms such as *E. coli* and *Klebsiella pneumoniae* are regarded as significant agents of environment-associated bovine mastitis [5]. *Yersinia enterocolitica* is the most prevalent *Yersinia* species connected to disease and acts as a causative agent of gastroenteritis [6].

Bacterial contamination also reaches groundwater by many routes; domestic and wild animals, birds, and farms wastes which present in a watershed area or within groundwater hydrological catchments [7]. Biomass, from degradable matters deposited into cattle water distribution pipes, accumulates biofilms, which improve the growth of bacteria and protect them against disinfectants [8].

Chlorine and chlorine-containing substances such as sodium hypochlorite (NaOCL) (bleach), chloramine T, chlorine dioxide, and isocyanurate are dissolved in water to sanitize or eliminate microorganisms. Many microorganisms have been found to develop resistance to different water disinfectants, including chlorination [9]. Sanchez-Vizuete *et al*. [10] and Bertelli *et al*. [11] reported numerous chlorine-resistant bacterial strains in drinking water.

There are several types of disinfectants that are used in the dairy farms; (sodium dichloroisocyanurate, NaOCL, hydrogen peroxide, peracetic acid, peroxymonosulfate, quaternary ammonium compounds [QACs], chloramine T, and chlorine dioxide) which are highly effective, easy to use, and stable for many purposes inside the farms [12]. However, the misuse of these disinfectants, including overuse, low doses use, lack of change, and other factors lead to the development of disinfectant microbial resistance. Among these disinfectants, QACs are widely used; this develop questions about the potential role of QACs in enhancing antimicrobial resistance development, mainly cross- or co-resistance to antibacterial agents [13].

Today, five QAC resistance genes (*qacE*, *qacEΔ1*, *qacF*, *qacG*, and *sugE* [p]) have been detected and identified on mobile genetic elements in Gram-negative bacteria. These resistance genes are related to small multidrug resistance family and are integron and/or plasmid-encoded, allowing efflux-mediated bacterial resistance against QACs [14]. The *qacE* gene is located mainly in 3’-CS of Class 1 integrons in Gram-negative microorganisms and the *qacEΔ1* gene is a deletion mutation of *qacE* which is the most widely spread gene found in QACs resistant *E. coli* strains [15], and has been related to a higher minimum inhibitory concentration (MIC) of benzalkonium chloride [16]. The *qacF* gene shows a high similarity degree (67.8% identity) to the *qacE* gene [17]. The *qacG* gene has been found in Class 1 integrons in Gram-negative microorganisms, while *sugE* (p) is frequently found on an *IncA*/C multidrug resistance plasmid that commonly found in Salmonellae [18].

The present study aimed to investigate drinking water bacterial profile, determine chlorine-resistant strains, and statistically correlate that with the used disinfectant and disinfection process variables. *In vitro*, evaluation of the bactericidal effect of the most commonly used disinfectants in cattle operations against the isolated chlorine-resistant strains and detection of *qacE* resistance gene in the isolated chlorine-resistant *Escherichia coli* strains in some cattle farms suffering coliform and non-coliform related disease around Egypt.

## Materials and Methods

### Ethical approval

Ethical approval is not applicable to this study.

### Informed consent

Informed consent was obtained from each participant.

### Field survey

#### Study area and period

A field study was conducted during the period from October 2016 to September 2018 in four districts, all over Egypt; West Delta (including Beheira, and Alex Desert Road), Middle Delta (including Menoufia, and Gharbia), East Delta (including Kaluobia, Sharkia, Dakahlia, Ismailia and Desert Road), and Upper Egypt (including Fayoum, Beni-Suef, and Minya). The selection criteria of survey farms were based on the previous history, obtained by questionnaire, of cattle health problems associated with drinking water in the investigated areas. From each farm, representative aleatory water samples were collected from water troughs in adult animal houses, at beef cattle farms (n=60), dairy cattle farms (n=60), and dairy beef mixed farms (n=12), total 132 farms under investigation.

#### Study design

It was a transversal study done using questionnaires. The protocol of the study involved two steps; in the first step, water samples were collected from cattle farms for isolation and identification of bacterial profile using both biochemical and serological techniques. A structured questionnaire was assembled to identify the associated hygienic risk factors in survey farms, such as the used disinfectants and disinfection processes. The obtained data were analyzed to identify the risks associated with the occurrence and spreading of different bacterial strains and resistant serotypes in cattle farms.

In the second step, water samples from drinkers were bacteriologically examined for total viable colony prior application and post-application of chlorine disinfectant for the detection of the chlorine-resistant strains. Finally, estimated efficacy of eight commonly used disinfectants against the isolated chlorine-resistant bacterial pathogens. Data were recorded and statistically analyzed.

#### Questionnaire survey

A structured questionnaire was prepared, including full farm identification and information regarding risk factors attributed to disinfection process. Recording disinfection attributes which mainly include both disinfectant type and frequency of use or change in general farm disinfection, floor disinfection, feeder disinfection, calf feeder disinfection, wheel dip, foot dip, hoof dip, and milk house including disinfectant types used in general milk house disinfection, teat dip, milk line, and milk tanks. All data were obtained from clinical records of the farm or interviews with the owners and veterinarians.

#### Cattle farms descriptions

In most of the studied dairy and large beef farms, the housing type is loose/free stalls in which the animals are kept in separate yards, and each yard is provided with manger and water trough located under sheds. The animals are left free in a yard with an area of about 7-10^2^ m/head. Yards were not provided with a drainage system resulting in accumulation of manure except only one closed farm, which keep cows in cubicle/free stalls. Water was always available, from public net, surface water, or underground pump, for the purposes of drinking, washing, and milking hygiene. The hygienic measures that prevailed in these farms were fair.

#### Water sampling

Water samples were collected equally in winter (December, January, and February) and summer (June, July, and August) seasons from all farms under investigation and comprising three sources: ground, surface, and commercial tap water.

The water samples were collected in two separate bottles, one for microbiological analysis and the other for selection of chlorine-resistant bacteria, clean, dry, sterilized screw-capped glass bottles of 1 L capacity previously sterilized in hot air oven at 170°C for 60 min were used, the bottles were rinsed several times with the water to be sampled before collection. Samples were stored at 4°C and analyzed within 48 h of sample collection as described by Kamal *et al*. [1].

At the same time, dipping of Dip-Slides (©Liofilchem®) into troughs water for further evaluation of water bacterial profile: (1) CONTACT SLIDE Chrom 2 (Chromatic™ Coli Coliform/Plate Count Agar+TTC+Neutralizing) Flex Dip-slide with a chromogenic selective medium for detection and enumeration of *E. coli* and coliforms and a non-selective medium for total bacterial count. (2) CONTACT SLIDE 5 (flexible slides for the detection and enumeration of *Enterobacteriaceae* and fecal Streptococci). (3) CONTACT SLIDE 4 (flexible slide for *Pseudomonas*, yeasts, and molds detection and enumeration) were used [19]. Each sample was labeled and identified showing its source, site, type of watering system, and date of sampling. All the collected samples were transferred to the laboratory within 2 h.

### Laboratory examination of water samples

#### Microbiological examination of water samples

1. Isolation and identification of different microbes to identify the microbial profile

Nutrient broth tubes were inoculated with 1 ml of each water sample and incubated at 37°C for 24 h then further plated using nutrient agar plates. Inoculated plates were incubated at 37°C for 24-48 h. Suspected colonies were picked up and subcultured on different selective media for further identification then, subjected to biochemical identification [20]. Furthermore, all bacterial isolates were confirmed biochemically using the analytical profile index 20E system (BioMerieux, Marcy-l’Etoile, France). The laboratory work was done in the laboratory of the Department of Veterinary Hygiene and Management, Faculty of Veterinary Medicine, Cairo University.

2. Dip-slides incubation and evaluation was done according to the manufacturer manual and technical sheet [21].

#### Selection of chlorine-resistant bacteria by chlorine treatment of water samples

Water samples were collected from different sites in each farm used directly in this step. Treatment of a 1-L of each water sample with 500 µg/L of NaOCL (4% w/v available chlorine) for 30 min, it was filtered and bacteria isolated. The isolated bacteria after chlorine treatment were identified [22].

#### Serological identification of the chlorine-resistant isolates

Chlorine-resistant isolates (*Salmonella* serovars, *E. coli*, and *Pseudomonas aeruginosa* strains), subjected to Serotyping in Animal Health Research Institute, Dokki, Giza.

Diagnostic *Salmonella* antisera

The isolated *Salmonella* strains were identified serologically using *Salmonella* antisera polyvalent (O) and separate (O) factors anti-sera, polyvalent (H) antisera, (H) factor antisera for Phase I and Phase 2 according to Kauffmann-white scheme [23].

Diagnostic *E. coli* antisera

The isolated *E. coli* were identified serologically to identify somatic antigen “O” using slide agglutination test by diagnostic (O) serogroups, which consisted of eight polyvalent groups and 43 monovalent serovars according to Edwards and Ewing [24].

Diagnostic *P. aeruginosa* anti-sera

The isolated *P. aeruginosa* was identified serologically to detect different serogroups of somatic antigen “O” using the diagnostic (O) serogroups, which consisted of three polyvalent groups and 14 monovalent serovars [25].

### *In vitro* experiment

Estimated the efficacy of eight commonly used disinfectants against the chlorine-resistant isolated strains.

Once bacteria had been tested for chlorine resistance, isolated, and identified, pure cultures of the chlorine-resistant strains (*E. coli*, *Salmonella*, *P. aeruginosa*, *Streptococcus faecalis*, and *Klebsiella*), isolated from the water samples were tested for evaluation of the bactericidal effect of the selected disinfectants on these strains. Selected disinfectants were representing three different groups of disinfectant active ingredients that most commonly used for drinking water sanitation. The disinfectants are halogens (Iodine, chlorine dioxide, isocyanuric acid, and chloramine T), oxidizing agent (hydrogen peroxide, peracetic acid, and peroxygen), and quats (QACs).

#### Disinfectant test method

The suspension test protocol used was based on the European standard for evaluating the bactericidal activity of chemical disinfectants and antiseptics (BS EN 1276:2009). This stringent standard test requirements and method were carried out [26] and require a large reduction in microbial count (at least 5 log) within 5 min contact time with the test substance at 20°C in both clean and dirty conditions. The neutralizing solution consisted of tryptone, sodium chloride, lecithin, Tween 80, sodium thiosulfate, L-histidine, and saponin. Interfering substance was bovine serum albumin at 0.30% w/v (dirty conditions). Samples were incubated at 20°C, using three contact time (1 min, 5 min, and 15 min) and different disinfectant dilutions [27].

### Polymerase chain reaction (PCR) amplification

The PCR method was performed to detect the *qacE* gene among four *E. coli* chlorine-resistant strains (code: E1, E2, E4, and E5). The primers used to amplify *qacE* (forward: 5´AAGTAATCGCAACATCCG 3´ reverse: 5´ CTACTACACCACTAACTATGAG 3´). The DNA template was prepared by suspending an overnight culture in 600 µl of reagent-grade water. The suspensions were heated at 100ºC for 10 min and centrifuged at 13,000× *g* for 5 min. Each 25 µl of PCR mixture consisted of 2 µl of template, 5 µl of 5× PCR buffer, 1.5 mM MgCl_2_, 200 mM deoxynucleotide triphosphates, 0.4 mM primers, and 1.25 U of polymerase (Promega, Madison, WI, USA). The PCR conditions were set as follows: 94°C for 5 min, followed by 30 cycles of 94°C for 30 s for denaturation, 53°C for 30 s for annealing, and 72°C for 50 s for extension. Finally, the PCR products were incubated at 72°C for 10 min. The positive control of PCR was DNA of *E. coli* strain previously sequenced as it shows positive *qacE* gene presence. The negative control of PCR was DNA of *E. coli* strain previously sequenced as it shows a negative *qacE* gene presence. Amplified PCR products were analyzed on a 1% agarose gel stained with ethidium bromide by electrophoresis and visualized under ultraviolet light [15].

### Statistical analysis

For analysis of data, Statistical Package for the Social Sciences software, version 25.0 (SPSS Inc., Chicago, IL, USA) was used. Initially, all information gathered through questionnaire was coded into variables. The normality of data was tested using Kolmogorov-Smirnov test. Both descriptive and inferential statistics involving Chi-square test, Mann–Whitney U-test, Kruskal–Wallis H test, and binary logistic regression were used to present results. The effect size was calculated by eta-squared value. For each test, p<0.05 was considered statistically significant [28].

## Results

The survey applied on 46 farms in West Delta (17 in Behira and 19 in Alex Desert Road), 12 farms in Middle Delta (6 in Menoufia and 6 in Gharbia), 52 farms in East Delta (6 in Kaluobia, 7 in Sharkia, 6 in Dakahlia, and 33 in Ismailia Desert Road), and 22 farms in Upper Egypt (16 in Fayoum and 6 in Beni-Suef and Minya).

The questionnaire survey, including 132 questionnaires collected one from each farm. They revealed the number of different disinfectant items and frequency of use or change with their percent in survey farms. Descriptive statistics for each item in the questionnaire are given in Table-1.

**Table-1:** Number (%) of the survey farms using each disinfectant type (T) and disinfectant frequency of use or change (F).

General disinfection (T)	Hoof dip disinfection (T)	General parlor (T)
No: 51 (38.6%)	No: 67 (50.8%)	No: 7 (9.7%)
Formalin: 26 (19.7%)	CuSO_4_: 38 (28.8%)	Iodine: 19 (26.4%)
Phenol: 4 (3%)	Formalin: 21 (15.9%)	NaOCl: 29 (40.3%)
NaOCl: 50 (37.9%)	CuSO_4_+ZnSO_4_: 5 (3.8%)	Peroxygen: 13 (18.1%)
Glutaraldehyde: 1 (0.8%)	Formalin+CuSO_4_: 1 (0.8%)	Peracetic: 2 (2.8%)
General disinfection (F)	Hoof dip disinfection (F)	H_2_O_2_: 2 (2.8%)
No: 51 (38.6%)	No: 67 (50.8%)	Teat dip (T)
Random: 36 (27.3%)	Per 250 cows: 8 (6.1%)	No: 7 (9.7%)
Monthly: 12 (9.1%)	Per 500 cows: 39 (29.5%)	Iodophor: 61 (84.7%)
Seasonal: 33 (25%)	Per 200 cows: 18 (13.6%)	NaOCl: 3 (4.2%)
Floor disinfection (T)	Feeders disinfection (T)	QACs: 1 (1.4)
No: 11 (8.3%)	No: 112 (84.8%)	Milk line (T)
Slaked lime: 97 (73.5%)	Slaked lime: 14 (10.6%)	No: 7 (9.7%)
Quick lime: 24 (18.2%)	Quick lime: 3 (2.3%)	NaOH+Nitric: 44 (61.1%)
Floor disinfection (F)	Formalin: 3 (2.3%)	NaOH+Nitric+NaOCl: 21 (29.2%)
No: 11 (8.3%)	Feeders disinfection (F)	Milk tanks (T)
Random: 35 (26.5%)	No: 112 (84.8%)	No: 7 (9.7%)
Monthly: 14 (10.6%)	Random: 4 (3%)	NaOCl: 48 (66.7%)
Seasonal: 62 (47%)	Annual: 3 (2.3%)	Peroxygen: 13 (18.1%)
Annual: 10 (7.6%)	Monthly: 12 (9.1%)	Peracetic: 1 (1.4%)
Wheel dip disinfection (T)	Seasonal: 1 (0.8%)	H_2_O_2_: 2 (2.8%)
No: 69 (52.3%)	Calf feeders disinfection (T)	QACs: 1 (1.4%)
Phenol: 43 (32.6%)	NaOCl: 75 (56.8%)	Overall disinfectants types count
Formalin: 20 (15.2%)	Iodine: 26 (19.7%)	One: 1 (0.8%)
Wheel dip disinfection (F)	Peroxygen: 13 (9.8%)	Two: 33 (25%)
No: 69 (52.3%)	Peracetic: 1 (0.8%)	Three: 25 (18.9%)
Weekly: 56 (42.4%)	H_2_O_2_: 2 (1.5%)	Four: 34 (25.8%)
Monthly: 7 (5.3%)	KMnO_4_: 14 (10.6%)	Five: 34 (25.8%)
Foot dip disinfection (T)	QACs: 1 (0.8%)	Six: 3 (2.3%)
No: 102 (77.3%)	Calf feeders disinfection (F)	Seven: 1 (0.8%)
Formalin: 4 (3%)	Daily: 33 (25%)	Eight: 1 (0.8%)
Phenol: 26 (19.7%)	Between calves: 41 (31.1%)	Overall disinfectants rate use
Foot dip disinfection (F)	Random: 46 (34.8%)	High: 52 (39.4%)
No: 102 (77.3%)	Weekly: 12 (9.1%)	Low: 80 (60.6%)
Daily: 20 (15.2%)		Overall disinfectants rate change
Weekly: 10 (7.6%)		High: 87 (65.9%)
		Low: 45 (34.1%)

No=Means that the farm does not use any disinfectant in this disinfection process. NaOCl=Sodium hypochlorite, QACs=Quaternary ammonium compounds

Microbiological analysis of the collected water samples from house drinkers of the surveyed farms, revealed various results for microbial profile analysis. Eleven microbes were isolated, identified, and the frequency of each microbe in farms is shown in Table-2.

**Table-2:** Frequency number (%) of farms from which each microbe was isolated and identified.

Microbial profile	

Microbial spp.	n (%)
*Escherichia coli*	130 (98.5)
*Streptococcus faecalis*	129 (97.7)
*Pseudomonas aeruginosa*	129 (97.7)
*Klebsiella* spp *.*	101 (76.5)
*Proteus* spp *.*	88 (66.7)
*Salmonella* spp *.*	48 (36.4)
*Enterobacter* spp *.*	104 (78.8)
*Citrobacter* spp *.*	98 (74.2)
*Shigella flexneri*	40 (30.3)
*Serratia marcescens*	39 (29.5)
*Yersinia enterocolitica*	19 (14.4)

Laboratory examination of water samples for the detection of pathogen bacteria resistant to chlorine revealed that 19 bacterial strains from 19 farms out of 132 farms with a confidence interval (CI) (0.08-0.20), showed resistance in chlorine resistance test, as shown in Table-3 which also shown the serotyping of these chlorine-resistant isolates.

**Table-3:** Chlorine-resistant strains serotyping with their serotype code.

Chlorine-resistant bacteria	Serotyping of chlorine-resistant strains	Serotype code
*Escherichia coli*	*Escherichia coli* (polyvalent VII) (monovalent O144)	E2
*Streptococcus faecalis*	*Streptococcus faecalis*	St4
*Pseudomonas aeruginosa*	*Pseudomonas aeruginosa* (polyvalent II) (Group M)	P1
*Salmonella* spp.	*Salmonella* Volta 11:r:1, Z_13_, Z_28_	S2
*Pseudomonas aeruginosa*	*Pseudomonas aeruginosa* (polyvalent II) (Group K)	P4
*Pseudomonas aeruginosa*	*Pseudomonas aeruginosa* (polyvalent II) (Group M)	P3
*Salmonella* spp.	*Salmonella* Montevideo 6, 7, 14:g, m [p], s: [1, 2, 7]	S3
*Streptococcus faecalis*	*Streptococcus faecalis*	St1
*Klebsiella* spp.	*Klebsiella pneumoniae*	K2
*Escherichia coli*	*Escherichia coli* (polyvalent IV) (monovalent O6)	E5
*Streptococcus faecalis*	*Streptococcus faecalis*	St2
*Pseudomonas aeruginosa*	*Pseudomonas aeruginosa* (polyvalent II) (Group J)	P2
*Streptococcus faecalis*	*Streptococcus faecalis*	St3
*Salmonella* spp.	*Salmonella arizonae*	S1
*Escherichia coli*	*Escherichia coli* (polyvalent III) (monovalent O158)	E1
*Klebsiella* spp.	*Klebsiella pneumoniae*	K1
*Salmonella* spp.	*Salmonella nitra* 2, 12:g, m:−	S4
*Escherichia coli*	*Escherichia coli* (polyvalent V) (monovalent O25)	E4
*Escherichia coli*	*Escherichia coli* (polyvalent IV) (monovalent O27)	E3

**Figure-1:**
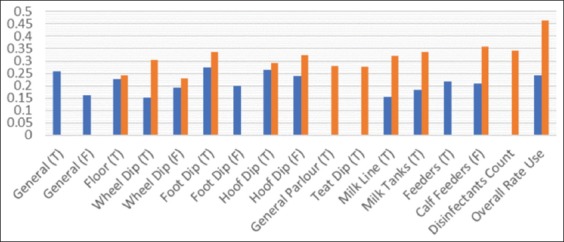
Spearman’s rho correlation coefficient between microbial profile count and presence of resistant strain, with particular disinfectant type (T) and disinfectant frequency of use or change (F).

For evaluation of the correlation, inferential statistics using spearman rank correlation revealed statistically significant correlation (p<0.05) between count of microbial strains in microbial profile of each farm (microbial profile count) and presence of resistant strains with particular disinfectants type (T) and disinfectant frequency of use or change (F), as shown in Figure-1 which shows Spearman’s rho correlation coefficient values.

Inferential statistics using Kruskal–Wallis H test to obtain mean ranks and calculate effect size by eta-squared measures of association to estimate the effect of disinfectants type (T) and disinfectant frequency of use or change (F) on bacterial profile count. The test shows that each particular disinfectant type (T) and frequency of use or change (F) affected the microbial profile count with specific mean ranks as shown in Table-4 which revealed mean ranks in descending order and eta-squared of each disinfection type and frequency.

**Table-4:** Impact of disinfection and disinfectants on microbial profile count, eta-squared measures of association of each disinfection type and frequency, and mean ranks in descending order of each disinfectant type (T) and frequency (F).

General (T) (eta=0.142)	Hoof dip (T) (eta=0.141)	Milk line (T) (eta=0.13)
Formalin: (88.19)	Formalin+CuSO_4_: (98)	No: (84.29)
No: (66.98)	Formalin: (83.57)	NaOH+Nitric acid: (68.84)
NaOCl: (59.19)	No: (72.79)	NaOH+Nitric acid
Phenol: (24)	CuSO_4_+ZnSO_4_: (49.2)	+NaOCl: (41.52)
Glutaraldehyde: (13.5)	CuSO_4_: (47.42)	Milk tanks (T) (eta=0.103)
General (F) (eta=0.045)	Hoof dip (F) (eta=0.126)	No: (84.29)
Seasonal: (75.76)	No: (72.79)	H_2_O_2_: (82.5)
No: (66.98)	Per 500 cows: (70.49)	NaOCl: (65.49)
Random: (64.43)	Per 200 cows: (44.56)	Peroxygen: (41.88)
Monthly: (45.21)	Per 250 cows: (43.75)	QACs: (34.5)
Wheel dip (T) (eta=0.059)	Foot dip (T) (eta=0.152)	Peracetic acid: (13.5)
No: (72.41)	No: (73.7)	Feeders (T) (eta=0.164)
Formalin: (67.88)	Formalin: (45.5)	No: (71.83)
Phenol: (56.37)	Phenol: (41.5)	Slaked lime: (39.86)
Wheel dip (F) (eta=0.055)	Foot dip (F) (eta=0.155)	Formalin: (31.33)
Monthly: (75.21)	No: (73.7)	Quick lime: (27.17)
No: (72.41)	Weekly: (51.6)	Calf feeders (F) (eta=0.106)
Weekly: (58.13)	Daily: (37.25)	Daily: (75.35)
	Floor (T) (eta=0.089)	Random: (74.26)
	Quick lime: (87.48)	Weekly: (65.88)
	No: (69.41)	Between calves: (50.85)
	Slaked lime: (60.98)	

No=Means that the farm does not use any disinfectant in this disinfection process. NaOCl=Sodium hypochlorite, QACs=Quaternary ammonium compounds

For evaluation of the effect of disinfectants rate of use on microbial profile count, inferential statistics using Mann–Whitney U-test to obtain mean ranks and calculate effect size by eta-squared measures of association were done. The test shows that disinfectants use rate affects the microbial profile count with mean ranks 73.71 and 55.41 for low and high use, respectively, with eta-squared 0.076.

For evaluation of the effect of each disinfectant type (T) and disinfectant frequency of use or change (F) on the presence of resistant strains, inferential statistics using Chi-square test to obtain cross-tabulation and Pearson Chi-square value were done. The test shows that particular disinfectant types (T) and frequency of use or change (F) significantly affect the presence of resistant strains with percent and Chi-square values, as shown in Table-5, and the percent presented in descending order.

**Table-5:** Chi-square value and percent of resistant strains presence in descending order for each significant disinfectant type (T) and frequency (F) of use or change.

Floor (T) (Chi=8.008)	Hoof dip (T) (Chi=19.858)	Teat dip (T) (Chi=17.549)
Slaked lime (19.6%)	Formalin+CuSO_4_ (100%)	Iodophor (27.9%)
No (0)	CuSO_4_+ZnSO_4_ (40%)	No (14.3%)
Quick lime (0)	Formalin (23.8%)	NaOCl (0)
Wheel dip (T) (Chi=24.04)	CuSO_4_ (23.7%)	QACs (0)
Phenol (34.9%)	No (3%)	Milk line (T) (Chi=16.48)
Formalin (15%)	Hoof dip (F) (Chi=18.59)	NaOH+nitric acid+NaOCl (33.3%)
No (1.4%)	Per 250 cows (50%)	NaOH+nitric acid (22.7%)
Wheel dip (F) (Chi=20.964)	Per 500 cows (23.1%)	No (14.3%)
Monthly (42.9%)	Per 200 cows (22.2%)	Milk tanks (T) (Chi=17.857)
Weekly (26.8%)	No (3%)	NaOCl (29.2%)
No (1.4%)	General parlor (T) (Chi=19.36)	Peroxygen (23.1%)
Foot dip (T) (Chi=15.463)	Iodine (36.8%)	No (14.3%)
Phenol (38.5%)	NaOCl (24.1%)	Peracetic acid (0)
No (8.8%)	Peroxygen (23.1%)	H_2_O_2_ (0)
Formalin (0)	No (14.3%)	QACs (0)
Calf feeders (F) (Chi=18.359)	Peracetic acid (0)	Disinfectants rate use (Chi=28.473)
Between calves (29.3%)	H_2_O_2_ (0)	High (34.6%)
Daily (21.2%)		Low (1.3%)
Random (0)		
Weekly (0)		

No=Means that the farm does not use any disinfectant in this disinfection process. NaOCl=Sodium hypochlorite, QACs=Quaternary ammonium compounds

For evaluation of the effect of the used disinfectants count on the presence of resistant strains, inferential statistics using binary logistic regression with entering method to obtain standardized coefficient (beta) were done. The test revealed that disinfectant count affects the presence of resistant strains with beta=0.808 and R^2^=0.189.

Results of *in vitro* experiment for evaluation of the bactericidal effect of the selected disinfectants on the chlorine-resistant isolated strains revealed the different effects of the used eight disinfectants with different contact times on the isolated chlorine-resistant strains, as shown in Table-6.

**Table-6:** Code of chlorine-resistant serotypes killed after 1 min, 5 min, and 15 min contact type with eight different disinfectants.

Disinfectant type	Code of serotypes killed after		

	1 min	5 min	15 min
Halogens			
Iodine	St2	O4, St1, St2, St3, St4	O4, St1, St2, St3, St4
Chlorine dioxide	St2	O4, P4, St1, St2, St3, St4	O4, O5, S3, P1, P3, P4, St1, St2, St3, St4
Isocyanuric acid	St2, St4	O4, S3, P3, P4, St1, St2, St3, St4	O2, O3, O4, O5, S3, P1, P3, P4, St1, St2, St3, St4
Chloramine T	St1, St2, St3, St4	O4, O5, S3, P1, P3, P4, St1, St2, St3, St4	O2, O3, O4, O5, S1, S2, S3, S4, P1, P3, P4, K1, St1, St2, St3, St4
Oxidizing agent			
Hydrogen peroxide	O4, P3, P4, St1, St2, St3, St4	O2, O3, O4, O5, S3, S4, P1, P3, P4, St1, St2, St3, St4	O1, O2, O3, O4, O5, S1, S2, S3, S4, P1, P3, P4, K1, St1, St2, St3, St4
Peracetic acid	O4, O5, S3, P1, P3, P4, St1, St2, St3, St4	O2, O3, O4, O5, S1, S2, S3, S4, P1, P3, P4, K1, St1, St2, St3, St4	O1, O2, O3, O4, O5, S1, S2, S3, S4, P1, P2, P3, P4, K1, St1, St2, St3, St4
Peroxymonosulfate	O2, O3, O4, O5, S2, S3, S4, P1, P3, P4, St1, St2, St3, St4	O1, O2, O3, O4, O5, S1, S2, S3, S4, P1, P3, P4, K1, St1, St2, St3, St4	O1, O2, O3, O4, O5, S1, S2, S3, S4, P1, P2, P3, P4, K1, K2, St1, St2, St3, St4
Quats (QACs)	Zero	St2, St3, St4	St2, St3, St4

QACs=Quaternary ammonium compounds

### Presence of qacE gene

**Figure-2:**
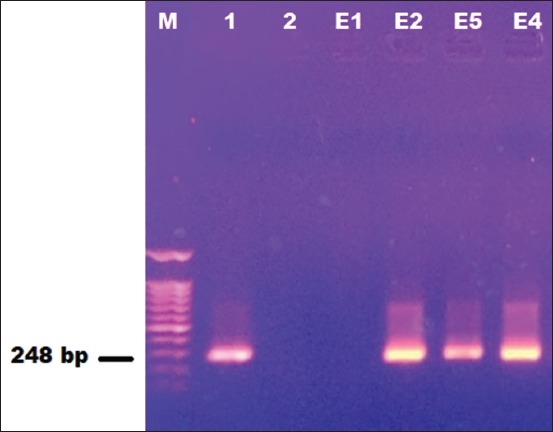
The presence of *qacE* gene among four *E. coli* chlorine-resistant strains. Lane M=100 bp ladder, lane 1=positive control, lane 2=negative control, lane E1=sample code O1 had negative result, lane E2=sample code O2 had positive result, lane E3=sample code O5 had positive result, and lane E4=sample code O4 had positive result.

To estimate the percentage of *qacE* resistance gene in selected *E. coli* strains isolated from cattle drinking water troughs, 75% (3/4) of the strains contain the *qacE* resistance gene with CI (0.33-1.17) and their PCR product, as shown in Figure-2.

## Discussion

Drinking water is considered as an important nutrient for livestock health and production, but prone to different microbial contamination by many factors which affect both health and performance of cattle [1]. The present study has focused on identifying the risk associated with the occurrence and spreading of different microbial strains, the most commonly used disinfectants, disinfection programs, and disinfectant resistant serotypes in cattle farms. Then, evaluating the efficacy of eight types of disinfectants against isolated bacterial pathogens which confirmed to be chlorine resistance and detect one of the most important genes (*qacE*) responsible for QACs disinfectant resistance in some isolates.

The results of this study in Egyptian cattle farms (Table-1) indicate that a wide range of disinfection types used for variable purposes inside cattle farms with a different frequency of use. For general disinfection, NaOCL was the most used disinfectant due to its cheap price and non-toxic effect, but over-use help in the development of bacterial resistance mainly with the high random frequency use; glutaraldehyde was the lowest used one due to high cost and toxic effect. For floor disinfection, slaked lime used with a high rate due to its low cost, easy handling, and drying effect, which encourages its use seasonally mainly in wet winter. For wheel and foot dip, phenol was used rather than formalin mostly due to phenol’s rapid long-acting effect with mild non-irritating odor. Foot dip was mainly changed daily for controlling individual movement but found that wheel dip was changed most weekly, which not recommended, may due to its large size and difficult to be renewed. For hoof dipping, many forms of disinfectants used but, copper sulfate was the highest may due to easy handling, low cost, and prolonged action. Hoof dip mainly changed per 500 cows in 29.5% of farms. Feeder disinfection was not applied in the majority of farms (84.8%) in adults house but applied mainly in calves house mainly with NaOCL in 56.8% of farms due to its commercial low-cost availability but this giving chance for chlorine resistance development, followed by iodine and potassium permanganate which also effective, safe and has low rate of bacterial resistance. In milk parlor, NaOCL mainly used for general parlor and milk tanks disinfection due to the previously mentioned causes, Iodophor used mainly in teat dip due to safety, effectiveness, and low price but the increasing misuse (heavy use and low change rate) leading to the development of microbial resistance. For milk line washing and disinfection, 61.1% of farms use sodium hydroxide for the alkaline cycle to remove organic matter and nitric acid in the acidic cycle for removal of mineral deposits. These recorded disinfectant types were in accordance with Fuqua [12] and USDA APHIS [29] which record the same types used in cattle farms operations with many variable frequencies.

With recording disinfectants rate of use in all farms, found that 60.6% of farms use a low rate of disinfectants (< 4 disinfectants) and with counting disinfectants rate of change from time to time in the same farms, found that 65.9% of farms make a high rate of disinfectant change. However, the majority of farms used a low rate of disinfectants with a high rate of change, the chlorine-resistant strains isolated from farms that use high rate of disinfectants with a low rate of change. These data lead us to hypothesize that farms that use a high rate of disinfectants with a low rate of change (i.e., disinfectants misuse) may develop a high rate of disinfectant resistant microbes [8,9].

The microbiological analysis revealed that the highest bacterial species isolated from all drinking water samples were *E. coli* in 98.5% of farms, these findings were in harmony with Fairbrother and Nadeau [30] and LeJeune and Wetzel [31] who found that *E. coli* was the most predominant bacterial contaminants in water of dairy farms. Meanwhile, drinking water in livestock farms contaminated with manure became a nidus for general bacterial growth leading to animal diseases [32]. Followed by *S. faecalis*, *P. aeruginosa*, *Enterobacter* spp., *Klebsiella* spp., *Citrobacter* spp., *Proteus* spp., *Salmonella* spp., *Shigella flexneri*, *Serratia marcescens*, and *Y. enterocolitica* in 97.7, 97.7, 78.8, 76.5, 74.2, 66.7, 36.4, 30.3, 29.5, and 14.4% of samples, respectively, as shown in Table-2 and these results are in accordance with different authors [3,7,33,34].

Chlorine-resistance test revealed that five *E. coli* strains (code: E1, E2, E3, E4, and E5), four *Salmonella* strains (code: S1, S2, S3, and S4), four *Pseudomonas* strains (code: P1, P2, P3, and P4), two *Klebsiella* strains (code: K1, and K2), and four *Streptococcus* strains (St1, St2, St3, and St4) show chlorine resistance, then subsequent serotyping was done for further identification as shown in Table-3. Study findings are similar to Ameh *et al*. [9] and Sanchez-Vizuete *et al*. [10] who recorded a number of bacteria have been shown to develop resistance to different agents used for the treatment of water, including chlorination.

A study survey recorded number of microbial strains in each farm sample (microbial profile). Statistical analysis shows significant weak to moderate correlation (rho 0.151-0.273) between microbial profile count with disinfection types and frequency of use or change, as shown in Figure-1. To know which disinfection type has the highest effect on microbial profile count, Table-4 revealed that all disinfection types and frequencies nearly have effect size with eta-squared range (0.045-0.164) but, there is a critical difference in mean ranks of each disinfectant type and frequency [35,36]. Furthermore, the study findings revealed that disinfectants use rate affects the microbial profile count with effect size eta-squared 0.076, low use rate has the highest mean rank 73.71, i.e., low rate of disinfectants use cause increasing count of microbial strains in water than high use rate which resembles the findings of Sanchez-Vizuete *et al*. [10] and Chastre and Trouillet [37].

A study survey recorded the presence of resistant strains in some farm samples. Statistical analysis showed significant weak to moderate correlation (rho 0.229-0.464) with disinfection types and frequency, as shown in Figure-1. Table-5 revealed that disinfectants rate of use has the highest Chi-square 28.473, i.e., has the highest effect as a cause of resistance strains presence. However, floor disinfection type has the lowest Chi-square value 8.008, so it has the lowest effect on resistance. All other disinfection types and frequencies have Chi-square value range (15.463-24.04), and each value reflects its effect on resistance strains presence. Furthermore, each particular disinfectant type and frequency has its percent (%) of resistance strain presence. Hence, resistance strains presence statistically affected by disinfection process type, disinfectant type, and frequency. Furthermore, study findings revealed that count of disinfectants used in each farm affects the presence of resistant strains with beta=0.808, which means each one increase in count of the used disinfectants leads to 80.8% increase in the presence of resistant strains with R^2^=0.189. These results are in accordance with the results of Kalmokoff *et al*. [38], Kahlmeter *et al*. [39], and Langsrud *et al*. [40] who mention that the widespread use of biocides has led to concerns on the emergence of bacteria with reduced susceptibility to biocides and their potential role in the development of antimicrobial resistance in bacteria.

Estimated the efficacy of eight disinfectants on the isolated 19 chlorine-resistant strains with 3 contact times. To pass the test, disinfectant must achieve a five-log reduction in viable colony count after each contact time [41]. After 1 min, peroxymonosulfate kills the highest number of strains (14/19), followed by peracetic acid (10/19), H_2_O_2_ (7/19), chloramine T (4/19), isocyanuric acid (2/19), chlorine dioxide (1/19), iodine (1/19), and QAC (0/19). After 5 min, the same order of disinfectants kills 17, 16, 13, 10, 8, 6, 5,3/19 strains, respectively. After 15 min, also the same order of disinfectants kills 19, 18, 17, 16, 12, 10, 5, 3/19 strains, respectively. Peroxymonosulfate is a chlorine releasing and oxidizing disinfectant, which is broad-spectrum disinfectant, has rapid prolonged action, resists organic matter, and has a low rate of use due to high cost, so bacterial isolates showed a low rate of resistance against peroxymonosulfate that kill 19/19 strains after 15 min. QAC has surfactant effect with low disinfection effect due to the presence of a high rate of bacterial resistance so, QAC was the least effective one that kills only 3/19 strains even after 15 min contact time. Gasparini *et al*. [42] found that peroxymonosulfate is effective against *E. coli* at recommended concentration but, disagree with Moustafa *et al*. [43] who found that peroxymonosulfate with recommended concentration gave unsatisfactory results in their study. Furthermore, these results are in accordance with others [44-46].

On hypothesize that these bacterial strains often contain resistance genes against disinfectants. QACs are commonly used in different farm activities and water disinfection. This raises questions about the possible role of QACs in promoting the development of antimicrobial resistance, in particular, co- or cross-resistance to antimicrobials [13]. Most *E. coli* strains are part of the normal intestinal flora, but some strains, such as diarrheic *E. coli*, can cause enteric infections. Contamination of water with *E. coli* usually occurs in cattle farms. In general, drinking water troughs are major reservoirs of antimicrobial-resistant *E. coli*. *E. coli* isolates from water sources have been shown to exhibit a high MIC of QACs that were correlated with general disinfection resistance, while *qacE* and *qacEΔ1* genes are the most widespread genes found in QACs resistant *E. coli* strains [15]. The study results revealed confirmed the presence of *qacE* genes in three (code: E2, E4, and E5) out of four (code: E1, E2, E4, and E5) isolated chlorine-resistant *E. coli* strains as shown in PCR results (Figure-2).

## Conclusion

We could conclude that drinking water microbial profile strains and resistance to disinfectants are widely varied in cattle farms, and this variance depends on critical factors among which the disinfection process types used disinfectant types and frequency of disinfectants use or change.

Further investigation of the effect of water chemical quality on microbial profile and the presence of other resistance genes and figuring out the relationship between disinfectants and antibiotic resistance in the isolated strains are required.

## Authors’ Contributions

ZAMA, MAKh, and JE contributed to the conception, design, provision of field sample, and drafting the manuscript, MAK produced data and drafting the manuscript, MAK and MAKh contributed to the designing of the study as well as analysis and interpretation of the data. All authors contributed to the final editing and approval of the manuscript.
